# Implant Imaging: Perspectives of Nuclear Imaging in Implant, Biomaterial, and Stem Cell Research

**DOI:** 10.3390/bioengineering10050521

**Published:** 2023-04-25

**Authors:** Andras Polyak, Zita Képes, György Trencsényi

**Affiliations:** Division of Nuclear Medicine and Translational Imaging, Department of Medical Imaging, Faculty of Medicine, University of Debrecen, Nagyerdei St. 98, H-4032 Debrecen, Hungary

**Keywords:** implant, biomaterial, tissue engineering, bioengineering, stem cell, nanoparticles, liposomes, PET, SPECT, CT

## Abstract

Until now, very few efforts have been made to specifically trace, monitor, and visualize implantations, artificial organs, and bioengineered scaffolds for tissue engineering in vivo. While mainly X-Ray, CT, and MRI methods have been used for this purpose, the applications of more sensitive, quantitative, specific, radiotracer-based nuclear imaging techniques remain a challenge. As the need for biomaterials increases, so does the need for research tools to evaluate host responses. PET (positron emission tomography) and SPECT (single photon emission computer tomography) techniques are promising tools for the clinical translation of such regenerative medicine and tissue engineering efforts. These tracer-based methods offer unique and inevitable support, providing specific, quantitative, visual, non-invasive feedback on implanted biomaterials, devices, or transplanted cells. PET and SPECT can improve and accelerate these studies through biocompatibility, inertivity, and immune-response evaluations over long investigational periods at high sensitivities with low limits of detection. The wide range of radiopharmaceuticals, the newly developed specific bacteria, and the inflammation of specific or fibrosis-specific tracers as well as labeled individual nanomaterials can represent new, valuable tools for implant research. This review aims to summarize the opportunities of nuclear-imaging-supported implant research, including bone, fibrosis, bacteria, nanoparticle, and cell imaging, as well as the latest cutting-edge pretargeting methods.

## 1. Introduction

Single photon emission computed tomography (SPECT) and positron emission tomography (PET) represent the mainstream non-invasive diagnostic tools at hand in the field of nuclear medicine. Applying selective radiopharmaceuticals (radiotracers), SPECT, and PET purvey three dimensional (3D) visual and quantitative information on specific molecular targets. The success of nuclear molecular imaging in medicine and biomedical research is reinforced by its multifunctional capabilities and multidisciplinary nature. One of the greatest technological advancements was the combination of the mentioned nuclear–medical imaging systems with either conventional computed tomography (CT) or magnetic resonance imaging (MRI) modalities, rendering the possibility of the simultaneous acquisition of both anatomical/morphological and functional information. Preclinical research units utilize miniaturized equivalents of these hybrid systems including SPECT or PET composed of CT or, more recently, MRI (mini/µ SPECT/CT, PET/CT, SPECT/MRI, and PET/MRI hybrid devices) explicitly designed for the accomplishment of small animal investigations. The latest translational hybrid µSPECT/CT/MRI and µPET/CT/MRI devices merge the highest morphological contrasts of MRI or CT and the maximal sensitivity of real-time functional PET or SPECT imaging. With their aid, the quantitative, high-resolution monitoring and visualization of novel molecular targets or the biodistribution of potential drug candidates becomes feasible [1,2].

In nuclear imaging, various radiotracers are employed that are pharmaceutical drugs or biomolecule analogues labelled with radioactive isotopes. Since radiopharmaceuticals do not exert any effect on either the biochemical or physiological processes of the subject, the evaluated biological phenomena and mechanisms could be observed in a non-invasive manner. Currently, a vast array of radiopharmaceuticals targeting a wide spectrum of diseases and biomarkers are in routine clinical usage in medical centers worldwide. However, despite the broad availability of imaging tools, very little effort has been made thus far to apply them to the follow-up of implants. Nuclear imaging modalities would serve as a magic bullet in the non-invasive, in situ evaluation and in-depth understanding of the interactions of tissues and body fluids with biomaterials as well as medical devices. While post-implantation in vivo imaging is restricted mainly to MRI and CT, the integration of more sensitive, quantitative, specific radiotracer-based SPECT and PET assistance into existing clinical paths still remains a challenge.

In the present review, we provide a detailed overview of implant- and biomaterial-relevant imaging modalities and the spectrum of the currently available diagnostic radiopharmaceuticals. The review first presents the most authentic modality, bone imaging, followed by infection, inflammation, biofilm, and fibrosis imaging techniques and relevant radiotracers. In the second half, we present the latest state-of-the-art molecular imaging techniques that may be relevant in implant research, including cell and nanoparticle imaging options, pretargeting supported custom biomaterial tracking opportunities, and related radiotheranostic and radiotherapeutic tools.

## 2. Bone Imaging: Quantitative Feedback on Bone Tissue Regeneration, Resorption, and Osseointegration

An ample number of studies have been recently centered around bone (dental) implant research, investigating the chemical, physiological, and mechanical factors related to bone tissue regeneration, bone resorption, implant osseointegration, osseoconduction, and bone remodeling. Even though SPECT and PET make the non-invasive and quantitative characterization of mineralization—associated with the growth of osteoblasts and osteoblast-like cells—on biomaterials possible, only a limited number of nuclear medicinal investigations were executed to profoundly assess the aforementioned osseus processes. Due to the strong affinity of bisphosphonates to the growing hydroxyapatite crystals on bone’s surface, all kinds of bone-related post-implantation progressions can be characterized by routinely produced bone-specific tracers.

Bone was considered metabolically inactive until Chiewitz and Hevesy showed the increased phosphorus-32 (^32^P) concentrations in the skeleton of rats in 1935 [3]. Radionuclide based bone imaging was first reported by Fleming and his colleagues in 1961 [4]. In their study, strontium-85 (^85^Sr) was encountered to be localized preferentially in areas with increased osteoblastic activity rather than in normal bone. Adding to this, the suitability of the ^85^Sr scan as an index of bone repair was also confirmed [4]. Applying technetium-99m-labelled phosphates (^99m^Tc, polyphosphate, and pyrophosphate) for SPECT imaging, Subramanian and McAfee experienced improved tracer uptake in bones [5]. The key turning point in skeletal scintigraphy came with the introduction of ^99m^Tc-labeled diphosphonates. These radiopharmaceuticals are analogues of pyrophosphates, characterized by the replacement of the P-O-P bond by a P-C-P one [6]. Given their capability to strongly attach to calcium phosphate, diphosphonates are potent inhibitors of both the crystallization of calcium phosphate and the dissolution of hydroxyapatite crystals [7]. Until now, a considerable number of diphosphonates with distinct physiological properties have been proposed in nuclear skeletal imaging, making radiolabeled-diphosphonate-based bone scintigraphy a sensitive tool for deciphering alterations in skeletal blood flow and osteoblastic activity, for example, in malignant processes and infectious diseases [8,9]. Later, in the 1990s—with the emergence of whole-body PET scanners—phosphonates were partly replaced by their PET-equivalent ^18^F-sodium-fluoride Na [^18^F]F, which offered higher resolution images with enhanced contrast [10]. Following the diffusion of stable fluoride into the skeletal extracellular matrix, at least 99% of the whole-body fluoride content was exchanged with the hydroxyl groups of the hydroxyapatite crystals on bone’s surface to form fluorapatite. Similarly to phosphonates, the pharmacokinetic and the osseous uptake of Na [^18^F]F was dependent upon skeletal perfusion, the exposed hydroxyapatite surface, and renal excretion [6,11,12].

Regarding implant imaging, the influence of skeletal graft proteins on the activity of bone formation (osteogenic activity) was investigated at a preclinical level in small animal systems using [^99m^Tc]Tc-methylene diphosphonate ([^99m^Tc]Tc-MDP). The assessment of bone metabolism was accomplished 6 weeks post-implantation by the γ-counter-based measurement of the radioactivity of the harvested organ samples. In addition to the primary hypotheses, the applicability of [^99m^Tc]Tc-MDP as an indicator of the rate of bone formation was proved [13]. Furthermore, [^99m^Tc]Tc-MDP seems to be valuable in the evaluation of dynamic impairments in bone metabolism around osseointegrated titanium implants prone to mechanical stress [14] or treated with growth factor [15].

**Figure 1:**
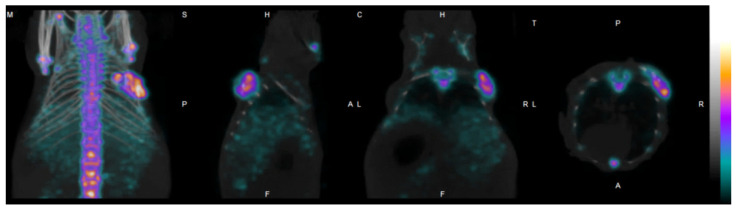
SPECT/CT imaging of bone metabolism on the surface of a bone tissue differentiated mesenchymal stem cell bearing hydrogel scaffold in mouse, employing the conventional radiopharmaceutical [^99m^Tc]Tc-MDP [16].

Exploiting the selective chemisorption of [^99m^Tc]Tc-MDP, the radiotracer was also applied in the in vitro quantitative assessment of the mineralization of osteoblast-like cells. For example, in a former study dealing with bone mineralization, after the selection of MC3T3 cells (osteoblast precursor cell) for culturing, the cultures were assessed both visually and quantitatively by gamma counting. In a similar vein, another study also strengthened the efficacy of [^99m^Tc]Tc-MDP in both the in vitro and in vivo quantification and identification of the mineralization of bone tissue derived from canine adipose mesenchymal stem cells (ADMSCs) [16]. In this study, the localization of bone-differentiated stem cells containing gel scaffolds were clearly visible with [^99m^Tc]Tc-MDP in in vivo mice xenografts (as presented in Figure 1). In addition—based on the number of synthesized minerals—the quantitative analysis revealed a significant radiotracer uptake in the slowly proliferating bone tissue.

## 3. Infection, Inflammation, Biofilm, and Fibrosis Imaging

Inflammation is the response to all kinds of tissue damage, caused by injury, surgical intervention, or invasion of micro-organisms [17]. In the latter case, the phenomenon is referred to as infection [17]. Taking into account that infection might occur without inflammation and inflammatory processes could take place without trauma, ischemia, neoplasm, foreign particles, device—or implant-induced microorganism influx—differentiation between bacterial infections, and aseptic inflammation became a major challenge in nuclear–medical diagnostics [17].

Inflammatory responses result in an increase in local blood supply and vascular permeability, along with the enhancement of plasma protein transudation as well as influx and glucose metabolism. Ex vivo radiolabeled inflammation-related leukocytes provide the basis for the SPECT imaging of inflammatory and infectious processes. Autologous leukocytes appended with indium-111-oxine ([^111^In]In-oxine, T_½_ = 2.8 days), later prelabelled technetium-99m-hexa-methyl-propylene-amine oxime ([^99m^Tc]Tc-HMPAO, T_½_ = 6 h), have been used for leukocyte scintigraphy since the 1970s [18,19,20]. Due to the half-lives of these nuclides, tracking times of several hours were possible. Prior literature data proved that infections of prosthetic vascular grafts and cardiac implantable devices could be identified with high sensitivities with the application of [^99m^Tc]Tc-HMPAO-tagged leukocytes [21,22]. In addition to precise lesion localization, SPECT/CT provides the opportunity to detect distant sites of diseases, for example, septic emboli [22,23]. Since infection triggers bone turnover in the hydroxyapatite matrix, the combination of leukocyte scans with radio-phosphonate-based three-phase bone scintigraphy (angiographic/perfusion, blood pool, and bone/delayed phase) in the diagnostic work-up of osteomyelitis was also employed [24].

Thereafter, enhanced glucose metabolism exhibited by the activated inflammatory cells was exploited as an alternative pathway to be imaged in the detection of infections. As the extent of inflammation is proportional to the rate of glucose consumption, accumulation of the radiolabeled glucose derivate ^18^F-labelled 2-fluoro-2-deoxy-glucose (2-[^18^F]F-FDG) allowed the PET-based detection of cellular glucose metabolism related to both inflammatory and neoplastic processes. Followed by the transmembrane glucose transporter (GLUT), catalyzed cellular uptake and phosphorylation by the hexokinase enzyme 2-[^18^F]F-FDG is captured inside the cell, meaning that it does not take part in further metabolic processes. As this intracellular metabolic trapping of [^18^F]F-FDG ensures the nuclear–medical imaging of glucose metabolism, nowadays, [^18^F]F-FDG embodies the most widely used, daily available gold standard radiopharmaceutical in nuclear medicine [17,25].

Extensive literature is available on the role of [^18^F]F-FDG PET/CT in diagnostic evaluation as well as the management of orthopaedic prosthetic implant infection [26,27]. The relatively low osseal tracer uptake along with an increased target-to-background ratio enables the precise identification of the inflamed regions [28]. In a study involving 215 patients with chronic osteomyelitis and orthopaedic implant-related infections, Wanter et al. reported that PET/CT had sensitivity specificities: a positive predictive value (PPV), a negative predictive value (NPV), and accuracies of 88%, 76%, 76% 89%, and 82%; respectively [27]. Given the high NPV of this imaging modality, unnecessary therapies and unwanted interventions could be avoided, which is pivotal regarding proper patient guidance. In addition, the tracer distribution pattern may have an impact on the choice of the appropriate surgical approach [28]. Due to semiquantification, [^18^F]F-FDG PET/CT could also be used for antimicrobial therapeutic response assessment, which is of critical importance from a clinical point of view [28]. Moreover, imaging with PET/CT is a potent alternative to MRI for those with metallic implants, as metalworks are not contraindicated for the performance of a PET/CT, and neither do they generate artefacts [29]. Hence, infections of vascular grafts, cardiac or implantable devices, and hip and knee protheses could also be successfully identified with mainstay [^18^F]F-FDG PET [30,31,32,33,34].

On the other hand, [^18^F]F-FDG PET may have limitations in inflammation imaging, including physiological heart and brain uptakes, background signals from blood, and interactions with blood glucose, requiring long fasting periods or special diets prior to imaging [35,36]. As a result, the use of radiotracers that selectively target inflammatory cells could greatly enhance the noninvasive detection of inflammatory activity. ^68^Ga-pentixafor is an agent that exhibits high affinity and selectivity for the C-X-C chemokine receptor type 4 (CXCR4), which is involved in various biological processes, including HIV entry, metastasis development, and autoimmune diseases such as rheumatoid arthritis, systemic lupus erythematosus, and multiple sclerosis [37,38] In addition to the detection of various tumors, ^68^Ga-pentixafor proved to be a promising candidate for detecting high-risk atherosclerotic plaques that exhibit intense inflammatory reactions and hypoxic conditions [36]. CXCR4 plays a crucial role in the trafficking of inflammatory cells, mediating the homing of progenitor cells in the bone marrow, regulating their mobilization into peripheral tissues upon injury [38]. Based on its overexpression cells involved in the inflammatory process, it was proved that ^68^Ga-pentixafor could potentially be used for detecting inflammatory cells [39,40,41,42,43] and infections [44].

By applying newly established PET radiotracers, bacterial infections could be accurately differentiated from sterile inflammations. For example, the groundbreaking translocator protein (TSPO) affine [^18^F]GE180 selectively indicates inflammatory processes (neutrophils and Ly6C^hi^ monocytes, shifting to macrophages) [45,46]. Regarding bacteria imaging, the detection of biofilm colonization on implants has been introduced. ^18^F-labelled maltohexaose derivate is one of the noteworthy biomolecules that selectively binds to the bacterial maltrodextrin transporter system that is not expressed by the mammalian cells; thus, it is able to uniquely indicate solely bacterial types of infections [47]. In an in vivo study conducted with a bifunctional bacteria-directed natural molecule—acting both as a ^64^Cu chelator and a specific ligand—demonstrated that this PET tracer is suitable for the visualization of particular bacterial populations, follow-ups of antibiotic treatment outcomes, and the tracking of bacteria in diverse biological niches [48].

**Figure 2:**
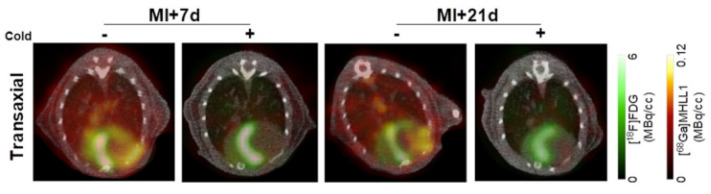
Fibrosis imaging: PET/CT fusion transaxial images at 7 d and 21 d after myocardial infarction with and without co-administration of unlabeled ligand. Accumulation of fibrosis tracer [^68^Ga]MHLL1 (orange) in the non-viable infarct territory defined by [^18^F]FDG (green) with additional distribution at the site of the surgical wound [52].

Post-inflammation tissue damage may result in the fibrotic transformation of the given tissues. Fibrosis is a wound-healing process that leads to tissue repair, remodeling, and, finally, scar tissue formation. Previous literature data note that the fibroblast activation protein (FAP)—expressed in the cell membrane during fibrotic scarring—is a potential biomarker for wound healing imaging. FAP is typically present during the process of rearranging the extracellular matrix, which means it can be detected in wound healing as well as non-cancerous diseases such as chronic inflammation, arthritis, fibrosis, and ischemic heart tissue following a heart attack. As a result, FAP inhibitors (known as FAPIs) have a strong affinities not only for tumors but also for various benign pathological conditions [49,50]. Performance evaluation studies reported that FAP-targeting FAPIs labelled with ^68^Ga are well-suited in the imaging of various fibrotic processes (as shown in Figure 2) [51,52,53,54,55,56,57]. Today, FAPI-PET/CT unquestionably presents a distinct opportunity for both diagnostic and therapeutic strategies in the management of tumors and certain benign conditions.

## 4. Cell and Stem Cell Tracking

Stem-cell-related applications have emerging potential in drug discovery and regenerative medicine. A rising number of imaging modalities enables the evaluation of the characteristics, differentiation, and the location of stem cells during therapeutic usage [58,59]. As remarked above, cell radiolabeling techniques—ranging back for many years of history in relation to infection and inflammation imaging—primarily applied ex vivo labelling procedures of autologous leukocytes mostly with prelabelled lipophilic molecules that were internalized by the cells through the cell membranes. Followed by the introduction of [^111^In]In-oxine and [^99m^Tc]Tc-HMPAO, several positron-emitter radionuclides have also been proposed for infection imaging, including short half-life gallium-68 (^68^Ga; T_½_ = 68 min) and fluorine-18 (^18^F; T_½_ = 110 min) [18,19,60,61,62,63,64]. Due to their half-lives, stem cell PET imaging of only short examination periods was possible using these tracers. Later on, copper-64 (^64^Cu) featured with a longer half-life (T_½_ = 12.7 h) began to emerge as a possible radiometal for radiolabeling procedures of stem cells [65,66,67]. ^64^Cu complexed with lipophilic tracers could be efficiently incorporated into the cells, albeit their rapid cellular efflux means a major constraint.

**Figure 3:**
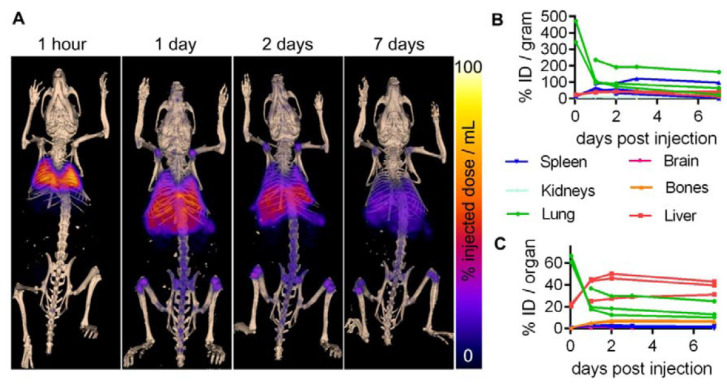
Whole-body biodistribution of ^89^Zr-oxine-labelled MSCTRAIL (tumor necrosis factor related apoptosis-inducing ligand – expressing mesenchymal stromal cells) followed up to 1 week post-implantation. (**A**) maximum intensity-projection PET showing ^89^Zr-oxine-labelled cells overlaid on 3D-rendered bone CT at the indicated time points after intravenous injection. (**B**) % injected dose (ID) per gram, using the wet weight of tissue from each animal following dissection at day 10 post-injection, and (**C**) % ID per organ [68].

Provided the similarly high lipophilic character of the oxinates of the transition radiometals ^68^Ga and ^111^In, such as [^68^Ga]Ga-Oxinate_3_ and [^111^In]In-oxinate_3_, these radiopharmaceuticals are also able to easily penetrate the cell membranes. Furthermore, the oxinate of zirzonium-89 (^89^Zr, T_½_ = 78.4 h)—[^89^Zr]Zr(oxinate)_4_—has recently arose as a successful prelabelled agent for rapid cell and liposome labelling under neutral conditions. The long half-life of ^89^Zr, offering a biologically more meaningful prolonged cell tracking time (even one or two weeks), deserves a special mention. Given its widespread commercial availability, [^89^Zr]Zr(oxinate)_4_ was already used for the labelling of the following various cell types: tumor cell lines, bone marrow and dendritic cells, therapeutic T cells, and stem cells (seen in Figure 3) [68,69,70,71,72,73,74,75,76,77,78,79].

When selecting the appropriate isotope, the half-life of the nuclides must be taken into consideration (in addition to the imaging modality), as the tracking time is limited, especially in the case of short half-life isotopes (^68^Ga). Although cell radiation exposure is negligible in such preclinical studies, it is important to always consider that the radioactive labeling procedure itself does not potentially affect the cell viability and function (remains truly non-invasive). It is important to also verify the stability of cell labeling after the prelabeled agents’ internalization.

## 5. Nanoparticle Imaging: Tracking Drug Delivery Systems and Drug Release

**Figure 4:**
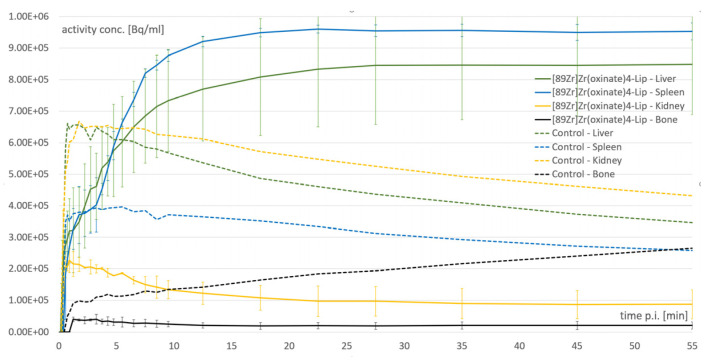
Real-time evaluation opportunity of nanovesicles post-injection: time-activity biodistribution curves of selected organs (liver, spleen, kidney, and bone) of mice generated from dynamic PET acquisition over one hour after i.v. application of zirconium-89-labeled liposomes [71].

Constantly evolving research and the industrial development of nanomaterials warrant the urgent need for the establishment of precise in vivo imaging systems. Given the ever-increasing spread of theranostics, comparable drug carrier systems—well suited for imaging—are advised to be proposed for the characterization, follow-up, regulation, optimization, and assessment of the pharmacokinetics of drug delivery [1]. For the realization of the future vision of image-guided regenerative therapies, bespoke medicine will require the careful matching of imaging with therapy. Currently, a broad set of chemical tools are available for the integration of effective methods for the radiolabeling of novel, individual nanovesicles. Furthermore, the proper conjugation or internalization of radiometals onto or into organic or inorganic, solid or core–shell structured, hydrophilic polymer (such as polyethylene glycol/PEG)-covered nanoparticles, or mono- and multilamellar lipid nanoparticles (NPs) could be accomplished with the easily accessible various types of bifunctional chelators (BFCs). This way, the real-time investigation of the path of nanovesicles is ensured post tracer administration (Figure 4). After dynamic PET and SPECT acquisition, organ uptake values, ex vivo biodistribution, and pharmacokinetic parameters of the labelled nanovesicles could be registered, including blood clearance or excretion times. The combination of a nuclear–medical imaging tool with CT or MRI modalities has a clear added value since these radiological tools ensure the precise localization of radiopharmaceutical accumulation. Consequently, in addition to ex vivo quantitative radioactivity measurements, the anatomical information of tracer accumulation could also be recorded with the application of SPECT/CT and/or PET/CT hybrid devices.

The following critical issues of nanosystem’s characteristics might be uncovered by PET: potential non-optimal PEG shell (too high “lost” part of particle fraction in the RES organs), inappropriately high particle size (due to aggregation), particle fraction polydispersity, or potential disintegration of the particles (early activity excretion via the kidneys and urinary tract). Furthermore, radiolabeling-based imaging might have the potential to sensitively project the effects of target-specific external stimuli such as light or magnetic fields on the investigated biomolecules, even prior to preclinical experiments [1]. The effective radioconjugation of liposomes, or organic and inorganic nanoparticles, could be executed by selecting either the BFC derivate suitable for the particle surface (shell) and the radiometal [1,71,80,81].

## 6. Pretargeting-Based Custom Biomaterial Imaging

Within the framework of pretargeted imaging, first, an unlabeled molecule—either receptor compounds or monoclonal antibodies—is delivered to the investigated tissue site, which could then be selectively targeted by a radiolabeled effector molecule characterized by high affinity to the receptor/antibody in vivo [82]. This two-step approach allows immediate and improved radiotracer accumulation at the targeted site (e.g., tumor tissue or potential implant), resulting in better contrast (tumor-to-background ratio) and a minor collateral radiation effect on the neighboring healthy tissues compared to the conventional targeting procedure [83]. Thus, pre-conjugated receptor molecules on biomaterials offer reproducible pretargeted imaging with rapid effector (tracer) pharmacokinetics and fast whole-body clearance of the radioactivity. The detailed method was successfully applied in several tumor-related studies that employed the noncovalent interactions between streptavidin and biotin and bispecific antibodies with radiolabeled effector hapten and oligonucleotide systems [84,85,86,87,88,89,90,91,92,93,94,95,96,97,98,99].

Click chemistry [100] enabled further exceptionally selective pretargeted imaging studies. Nuclear-imaging-related click chemistry is a modular approach in which a prelabeled agent (prosthetic group or BCF) binds to a biomolecule (e.g., a peptide or antibody) in vivo by a robust and selective click reaction. First, Tornøe et al. reported the use of a copper-catalyzed azide-alkyne cycloaddition (CuAAC) for the synthesis of a range of radiolabeled compounds, including ^18^F-labeled fluoro-alkylazides and ^11^C-labeled alkynes [101]. Later, more examples were published involving a wider range of SPECT radionuclides (^99m^Tc) and further PET isotopes (^68^Ga, ^64^Cu, ^89^Zr) [82,93,102,103,104,105].

Importantly, these methods require unique biochemical and radiochemical design and development, which, inevitably, impact experimental planning and costs. In such cases, preclinical studies should be preceded by more complex in vitro investigations (concerning radiochemical stability, specificity, selectivity, etc.) to exclude potential false negatives and false positives in vivo isotopic accumulations.

## 7. Radiotheranostic Opportunities

Radionuclide therapy (RNT, RT) or endoradiotherpy (ERT) is a form of treatment that uses alpha or beta emitter radionuclides to selectively deliver radiation to specific tissues or cells in the body for therapeutic purposes. In contrast to conventional external beam therapies, targeted radionuclide therapy is a non-invasive approach that minimizes collateral damage to normal tissues and delivers more specific radiation to tumors or inflamed lesions [106].

Taking the above into consideration, another possibility could be the incorporation of radiotheranostic solutions. Multifunctional nanoparticles offer diverse theranostic possibilities by conjugating theranostic isotope pairs [1], forming beta emitters (lutetium-177, yttrium-90, holmium-166) or alpha-emitters (radium-223, actinium-225), labeled therapeutic equivalents of their diagnostic variants [107].

Pentixafor and FAPI radiotracers mentioned in relation to inflammations may also be considered in a theranostic approach. Pentixather is a closely related alternative to Pentixafor, and, as its theranostic pair, it was selected for the development of the first CXCR4-targeted ^177^Lu-labeled endoradiotherapeutic agent [108]. The effectiveness of the theranostic strategy with a ^177^Lu-pentioxather, followed by a ^90^Y-pentixather, was demonstrated in mouse models and also in clinical examples [109,110,111]. The radiotheranostic FAPI approach represented a crucial step towards in the recent years. Thus far, there have been reports on over 100 patients who have undergone various FAP-targeted oncological radionuclide therapies, applying ^177^Lu- and ^90^Y-labeled FAPI agents [112,113,114,115,116,117].

## 8. Conclusions

As post-implantation in vivo imaging is mainly restricted to traditional MRI and CT modalities, the implementation of more sensitive, quantitative, specific radiotracer-based SPECT and PET methods in implant imaging still remains a challenge. Given that hybrid PET/CT and SPECT/CT systems purvey specific, quantitative, visual, and non-invasive feedbacks on implanted biomaterials, devices, or transplanted cells beyond mere anatomical data—provided by CT and/or MRI—the application of such devices would represent a unique and irreplaceable tracing modality in the establishment of sustainable improvements in the field of implant imaging. Furthermore, PET and SPECT techniques offer real-time in vivo observations with prolonged investigational times at high sensitivities, supporting the evaluation of the biocompatibility, inertness, and the immune response of different concerned biomaterials. The application of the wide range of regularly produced target-selective radiopharmaceuticals, the newly developed bacteria—inflammation—fibrosis-associated specific tracers, and radiolabeled individual nanomaterials, together with state-of-the-art imaging modalities (Table 1), may open a new era in implant imaging.

## Figures and Tables

**Table 1:** Some radiopharmaceuticals and labeling modalities available for implant-related studies.

Labeled Compound (Modality)	Mechanism	Promise	Limitations
2-[^18^F]FDG (PET)	indicates any enhanced tissue glucose metabolism	infection, inflammation	lower specificity, uptake in heart and brain, blood background signal, interaction with blood glucose
Na[^18^F]F (PET)	indicates enhanced bone metabolism	osseointegration, bone regeneration, bone resorption	lower specificity, lower imaging sensitivity compared to alternatives
[^68^Ga]FAPI (PET)	indicates fibrosis by targeting fibroblast activation protein (FAP)	inflammation, fibrosis, wound healing	limited specificity, inter-subject variability, limited availability, higher costs
[^18^F]GE180 (PET)	indicates inflammatory by translocator protein (TSPO)	inflammation	
^68^Ga-pentixafor (PET)	indicates C-X-C chemokine receptor type 4 (CXCR4)	inflammation	
^18^F-maltohexaose (PET)	specific for bacteria, indicates infections, allows to distinguish bacterial infections from sterile inflammations	infection, biofilms	limited specificity, antibiotics may preclude its application
^111^In-oxine-leukocytes (SPECT)	indicates infections and sterile inflammations by labeling of white blood cells	infection, inflammation	lower specificity, partly invasive
[^99m^Tc]Tc-HMPAO labeled and ^111^In-, ^68^Ga-, ^89^Zr-oxine labeled cells (SPECT or PET)	lipophilic prelabeled agent internalized by the cells	cell tracking	cell viability and function must be considered
^99m^Tc-labeled phosphonates, e.g., MDP (SPECT)	indicates enhanced bone metabolism	osseointegration, bone regeneration, bone resorption, infection	limited specificity, inflammation, infection, or healing fractures can increase the uptake
^18^F-, ^68^Ga-, ^89^Zr-, ^64^Cu-, ^99m^Tc-labeled nanoparticles (SPECT or PET)	direct tracking of nanocarriers	real-time tracking, evaluation of NP pharmacokinetics and biodistribution, imaging guided drug delivery, personalized medicine	limited tracing time depending on the selected radionuclide
pretargeted ligand, prelabeled agents (SPECT or PET)	selective pretargeting, click chemistry	specific imaging of preselected ligand, target	unique reactions, advanced bio- and radiochemistry
radiotheranostic agents (RNT, RT, ERT, theranosis, SPECT, PET)	switchable radioisotopes	imaging guided therapy, personalized medicine	availability, higher cost, advanced radiochemistry

## Data Availability

Not applicable.
